# Cyclodextrin-Enabled Polymer Composites for Packaging †

**DOI:** 10.3390/molecules23071556

**Published:** 2018-06-27

**Authors:** Lajos Szente, Éva Fenyvesi

**Affiliations:** CycloLab Cyclodextrin R & D Laboratory Ltd., H-1097 Budapest, Hungary; szente@cyclolab.hu

**Keywords:** aroma, barrier, biodegradability, films, food, flavor, fragrance, molecular encapsulation, plastic, preservatives

## Abstract

Cyclodextrin complexes of fragrances, antimicrobial agents, dyes, insecticides, UV-filters can be incorporated into polymers (packaging films, trays, containers) either to ensure the slow release or a homogeneous distribution of the complexed substances. This way the propagation of microorganisms on surface of enwrapped products is decelerated, or the product is made more attractive by slowly released fragrances, protected against UV-light-induced deterioration, oxidation, etc. Incorporating empty cyclodextrins into the packaging material an aroma barrier packaging is produced, which decelerates the loss of the aroma from the packaged food, prevents the penetration of undesired volatile pollutants from the environment, like components of exhaust gases, cigarette smoke, and reduces the migration of plasticizers, residual solvents and monomers, etc. Applying cyclodextrins in active packaging allows to preserve the quality of food and ensures a longer shelf-life for the packaged items.

## 1. Introduction

In contrary to the traditional packaging which serves merely as passive barrier designed to delay adverse effects of the environment on the packaged item, e.g., food, active packaging allows interaction between the packaged goods and the environment. The active packaging controls moisture, oxygen (modified atmosphere packaging), may contain antibacterial and/or antifungal agents to reduce food degradation and extend shelf life. The packaging material contacting food requires extra care in leaching the components (can diminish the release of plasticizers and other harmful compounds and control the release of the bioactive preservative components to maintain a low concentration for a long time).

This paper intends to give an overview on the application of cyclodextrins and cyclodextrin complexes in active packaging materials to show the usefulness of these cyclic carbohydrates in many roles improving the performance of packaging. The cyclodextrin-assisted packaging materials may contain “empty” cyclodextrins capable of entrapping migrating compounds. The other types of these materials contain cyclodextrins “filled” with different actives (these are inclusion complexes) thus providing protection against loss on heat during the preparation and a controlled-release of actives (e.g., antimicrobial, antioxidant, insecticide, animal and bird repellent compounds, etc.) in and around the packaging layers. The trigger for the release is humidity, making these materials especially useful for packaging the moisture-containing food items. Based on literature data and on CycloLab’s unpublished results this review shows both the possibilities and the limits of cyclodextrin applications in packaging materials, especially in active packaging.

### 1.1. Cyclodextrins Used for Packaging

**Figure 1:**
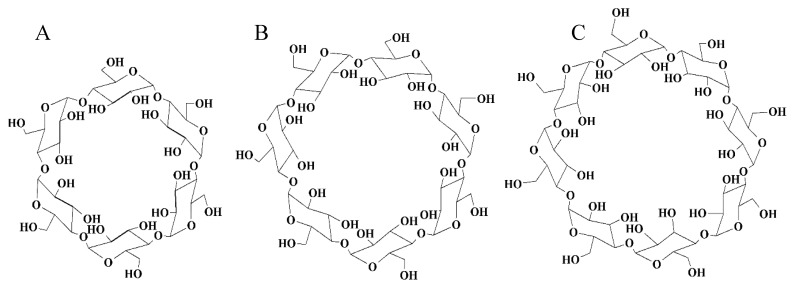
Schematic representation of the structure of alpha- (**A**) beta- (**B**) and gamma- (**C**) cyclodextrin.

More than 60,000 technical papers and patents are available on the structural and functional properties, production and utilization of cyclodextrins. The cyclic structure of the α-, β-, and γ-CDs (consisting of 6, 7 or 8 glucopyranose units, respectively) is shown in Figure 1 [1,2]. The use of cyclodextrins is spreading enormously for many reasons, such as [3]:

- They are semi-natural products; produced from a renewable natural material, starch, by a relatively simple enzymatic conversion.
- Their prices are low enough (in case of BCD < 10 USD/kg) to be acceptable for most industrial purposes.
- They are versatile in complexation of various molecules, and change important properties of the complexed substances significantly. The beneficiary effects of so called “molecular encapsulation” are widely utilized in industrial products, technologies, and analytical methods [4].
- The parent CDs are non-toxic taken orally and can be consumed by humans as ingredients of drugs, foods, or cosmetics.

The polar water molecules in the rather hydrophilic cavity of cyclodextrins are energetically disfavored (polar-apolar interaction), and therefore, can be readily substituted with appropriate guest molecules, which are less polar than water. This is the driving force of the complex formation [4,5].

Most frequently the host:guest ratio is 1:1, but 2:1 or 1:2 or other ratios have also been observed depending on the molecular structure of both the host and the guest.

The physical-chemical properties of the entrapped guest molecules are significantly influenced apparently, e.g.,:

- The diffusion and volatility (in the case of volatile substances) of the included guest can decrease strongly.
- The complexed substances, even gaseous substances can be entrapped in a carbohydrate matrix forming a microcrystalline or amorphous powder.
- The complexed substance can be effectively protected against heat decomposition, oxidation and any other type of reaction, except against those with the hydroxyl groups of cyclodextrin, or reactions catalyzed by them.
- The carbohydrate wrapping around the guest molecule makes the complex hydrophilic, easily wetted and rapidly soluble.

While biologically active substances complexed by cyclodextrin have been applied in various industries, such as in textiles [6], cosmetics, household and toiletry articles, pharmaceuticals [7], agriculture [1] and food [8,9], the present review is only dedicated to the application of cyclodextrins in packaging materials.

Cyclodextrins are safe in food. In the US the parent CDs are on the generally recognized as safe (GRAS) list. In Europe, α- and γ-CD can be consumed unlimitedly, no maximum acceptable daily intake (ADI) has been established, while ADI of 0.5 mg/kg body weight was allocated to β-CD [8,10]. The regulatory status suggests that the eventual migration of cyclodextrins into the food from the packaging has no harmful effects on consumers.

### 1.2. Polymer Packaging/Carrier Materials

A material used for packaging must have certain properties, such as:

- satisfactory tear strength,
- flexibility,
- low permeability for gases (O_2_, CO_2_), and low or higher permeability for water depending on the entrapped product,
- reduced UV-light transmission,
- low if any release of undesired components like monomers, softeners, plasticizers, etc.,
- appropriate compatibility with additives, such as pigments, antioxidants, or cyclodextrin complexes.

The most frequent packaging materials (films, cups, bottles, trays, sheets) are made of thermoplastic polyolefins (polyethylene, polypropylene, polybutylene), polyesters, or paperboard, or non-woven cellulose web, or poly(lactic/polybutyric acid).

The thermoplastic materials are frequently blended with other additives like sorbitan-monooleate, terephtalate, ethylene-vinyl acetate, vinylidene chloride, polyvinyl alcohol, etc. or coated with them on one or both sides. This way the excellent mechanical properties of the carrier material can be completed with the desired polarity (e.g., lower hydrophobicity). Another option is applying a hydrophilic coating layer (an alkyl-cellulose derivate, or chitosan), or the thermoplastic polymer can be laminated with a practically impermeable layer e.g., aluminum.

The packaging films, or containers: bottles, cups, trays, boxes, sheets, webs, etc. are usually produced from mono- or multi-component composites/laminates by extrusion. Nowadays the electrospinning technique has been introduced with a large surface of the resulting nanofibrous webs.

### 1.3. Incorporation of (Bio)active Components

Having selected both the carrier polymer, and the (bio)active component, the first question is how to get the latter incorporated into the polymer matrix. The second question is, how it will get released (if it is intended, because the UV filter or a dye should not migrate).

The packaging materials are usually produced at elevated temperature (over the melting point of the polymer, generally between 100 and 200 °C. This temperature is generally high enough to cause a considerable loss through volatilization, thermal conversion (degradation, isomerization) of the (bio)active ingredient. This loss can be avoided if the (bio)active component is mixed with the polymer in cyclodextrin-complexed form. A further advantage is the better homogeneity of the system, because majority of the (bio)active guests are not directly compatible with the polymer matrix. Sometimes, however, the cyclodextrin complex even when admixed as dry powder of very small particle size, forms larger aggregates in the polymer matrix. In such cases the (bio)active component has to be applied in the coating layer, which consists of a suspension of the cyclodextrin complex in a solution of a polymer compatible with the carrier film [11].

If the incorporated component has to exert its effect outside of the carrier-matrix, it has to get released in a controlled manner: this is the case with antimicrobial, fragrant, or plant hormone-like actives. If an incorporated fragrance, antimicrobial, insect repellent or plant hormone-like agent (all are rather lipophilic) is compatible with e.g., the very hydrophobic polyethylene, its release will be too slow and it cannot deliver the expected effect. The readily hydratable cyclodextrin helps in this case, as it creates hydrophilic micro-regions in the hydrophobic matrix, absorbing water, facilitating dissociation of the complex and creating channels for migration of the (bio)active guests. Further release-facilitating effects can be attained by incorporation of nanoclay into the polymer matrix [12].

Nevertheless, really good release profile for the matrix-incorporated (bio)actives can be attained only when the polymer matrix (or a coating layer containing the (bio)active compound) consists of a rather hydrophobic polymer e.g., poly(lactic acid), chitosan, or cellulose.

The low compatibility of the hydrophilic cyclodextrin and its complex with the less hydrophilic polymers results in impaired packaging material: reduced thermal stability, modulus of elasticity, tensile strength, elongation at break, and barrier capacity for water and oxygen [13]. This can be improved by using masterbatch. For instance, poly(lactic acid)-β-CD pellets with 30% β-CD content were applied to obtain films with suitable mechanical properties. Similarly, the mechanical and barrier properties of the packaging sheets were reduced at a lower extent by the use of masterbatch of the complex pre-blended with the polymer compared to the use of the crystalline complex directly [14].

Masterbatches of various cyclodextrin complexes combined with 50% low-density polyethylene have been marketed for active and smart packaging. These products can be added during plastic processing and the complex is “fused” into the plastic, this way enabling the plastic manufacturer to add fragrance, fungicide, pesticide, UV absorber, odorants, deodorants, and hundreds of other additives to plastic and other resin-based products. These masterbatches are compatible with a number of plastics including polypropylene, polyethylene, polyvinyl chloride, and ethylene/vinyl acetate copolymer.

Nowadays, the biodegradability of the packaging material has become of crucial importance. By incorporating cyclodextrin or its complexes, some weak (easy-to-degrade) points are built into the structure. It is even better if the carrier polymer is also biodegradable, such as poly(lactic acid), chitosan or cellulose. The high surface of the electrospun nanofibers of such biodegradable polymers ensures fast decomposition in the environment.

## 2. Cyclodextrin Complexes in Packaging Materials: Films, Laminates, Containers

**Figure 2:**
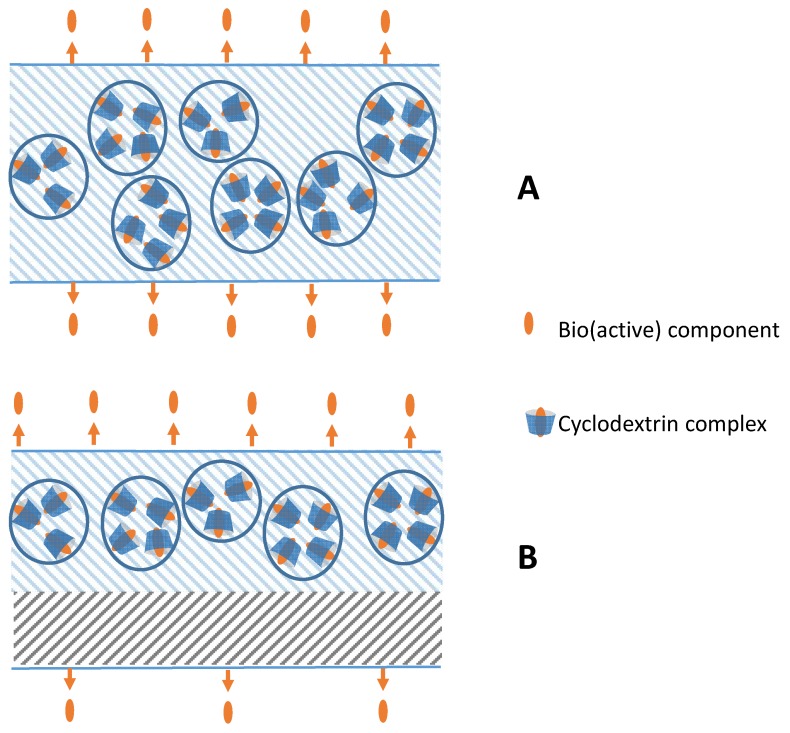
Cyclodextrin complexes are embedded into: (**A**) a polymer or (**B**) a coating layer on the polymer.

To attain or boost certain properties of the packaging materials, such as color, odor, antibacterial activity, permeability and light transmission, appropriate components are incorporated into the carrier polymer or into a separate coating layer on the polymeric matrix (Figure 2).

By mixing cyclodextrin complexes of fragrances, dyes, antimicrobial agents, insecticides, UV filter, etc. with thermoplastic polymers, various plastic products, such as films, containers, laminates and trays can be produced. In these products, the complexed substances are homogeneously dispersed and only slowly released from the polymer matrix [15]. The release rate depends on the concentration of the (bio)active compound in the polymer, its affinity towards the cyclodextrin, its diffusion coefficient in the polymer, its partition coefficient between the polymer and cyclodextrin and between the polymer and the packaged item, and also on temperature and time [16].

The powdered cyclodextrin complexes are prepared by reacting the cyclodextrin with the guest molecule in the presence of water, then removing the water (filtration + drying, spray-drying, freeze-drying). Before mixing it to the (generally pelleted) thermoplastic polymer, the complex has to be dried carefully, to remove even the strongly bound crystal water, otherwise it will escape at the melting temperature of the polymer matrix, resulting in foaming, and small “explosions”.

If the per se hydrophilic cyclodextrins or their complexes are incompatible with the selected polymer, appropriate “wetting” agents (e.g., poly(vinyl alcohol), glycerol) can be used, or instead of the natural cyclodextrin an appropriate cyclodextrin derivative (acetylated or methylated cyclodextrins, derivatives with long alkyl chain or sililated ones) may result in products of the desired properties. Table 1 illustrates a few examples on the variety of the applied cyclodextrins, included guest molecules, the material and the product form of the polymer matrix, and their field of application.

### 2.1. Fragrant Films

Cyclodextrin-complexed lavender or other perfume oils were mixed into a thermoplastic polymer and extruded into long-lasting fragrance plastic products [73,74]. Rosy smell fragrant cups, pen, cases, toys, etc. were produced adding “millet jelly” and geraniol complexed by cyclodextrin to polyethylene pellets before melting [75]. Transparent polyester sheets which contain 3% lemon oil complexed by maltosyl cyclodextrin had a lemon fragrance for at least 6 months [76].

A leather substitute emitting natural leather odor can be produced by incorporating the cyclodextrin-complexed leather perfume (comprising thymol, thyme oil, eugenol etc.), into molten polyvinyl chloride, and applied into leather substitute to be used e.g., in automobile carpets, door internal coverage, etc. [77,78].

Synthetic resin coated with a dispersion of cyclodextrin-stabilized perfume, and other (bio)active component has long-lasting effect [79]. The pages in a calendar printed with fruit design was coated with orange fragrance/β-CD complex to emit orange smell for about 3 months [80]. According to a Japanese patent perfume/β-CD complex applied in printing inks can be used for producing cards (e.g., telephone cards, business cards, bank cards) with long-lasting fragrance [81].

Animal and bird repellent plastics were developed, for instance, for use as plastic garbage bags [82].

### 2.2. Antimicrobial Packaging Materials

The antimicrobial effect is the most important in food packaging. For this purpose a few natural or analog synthetic antimicrobial agents are generally used, such as allyl isothiocyanate, hinokitiol or cedar leaf oil.

Freshness of fruits and vegetables can be maintained by hinokitiol and/or ethylene removers. For example, apples can be sealed into hinokitiol-impregnated plastic and cellulose films to keep freshness for an extended period [7,42,43,45].

Allyl isothiocyanate is a widely applied antimicrobial compound for food packaging as it is of natural origin (an organosulfur compound from mustard, radish, horseradish and wasabi). Its complex with β-CD can be incorporated into various plastic films and used for prolonging the shelf life of food [17,19,45,50,71,83]. Used as lining for egg containers it prevents the eggs to be infected with harmful microbes [20,21]. Incorporated in poly(lactic acid)-based (easily biodegradable) packaging material it was found to be useful for active packaging of hard cheese [12]. The rate of release ensured the necessary antimicrobial concentration in the immediate vicinity of the cheese surface (between the cheese surface and the packaging film) but not high enough for the penetration of significant amount of the fungicide into the cheese. A humidity-dependent release of allyl isothiocyanate was proved also from electrospun fibers from soy protein isolate/poly(ethylene oxide) blend and poly(lactic acid) packaging films containing the active compound complexed by β-CD [84].

Banana wrapped with poly(vinyl chloride) foil containing imazalyl/β-CD preserved its freshness for 12 days when stored at 13 °C, while the banana wrapped in commercially available poly(vinyl chloride) foil showed brown spots, the first signs of deterioration, when stored under the same conditions [83].

The β-CD complex of 2-methoxycinnamaldehyde has been incorporated into shoe insoles to inhibit the microbial growth and foul odors. Cotton fabric was immersed into ethanol-water solution containing 2-methoxycinnamaldehyde and β-cyclodextrin to retain 10 g active ingredient per square meter. This fabric was placed between two poly(vinylidene chloride) sheets. Conditions as athlete’s foot and symptoms such as rash, blisters, and skin drying were effectively treated.

Electrospun microfibrous webs containing triclosan/CD complex and poly(lactic acid) showed higher antimicrobial activity than the webs prepared similarly from uncomplexed triclosan and poly(lactic acid) [69].

### 2.3. Insecticidal Films

Citronella (a natural insecticide) complexed by cyclodextrin in coating of cardboard packaging can efficiently hinder the invasion of muesli and wheat germ by red flour beetles [85]. Pyrethroids or organic phosphates were complexed with cyclodextrins, mixed with polymers and shaped into plant containers, for protection of plants from insecticidal damage. For example, α-CD complex of fenitrothion was incorporated into ethylene-vinyl acetate copolymer, mixed, fused, and shaped into plant container boxes [86]. Another patent describes a process where microcrystalline fenitrothion/α-CD complex was mixed with soft poly(vinyl chloride), fused and pelletized [87]. By extruding the molten pellets to a sheet, insect- and mite-controlling carpet underlays were produced. Similarly, construction and furniture materials with insecticide properties can be also prepared.

Sustained-release insecticides, air fresheners, deodorants etc. can be produced in forms of sheet, tape or fibers using the fused mixture of a plastic material (polyethylene), a water-absorbing polymer (acrylic acid-vinyl alcohol copolymer) and the cyclodextrin complex of the active agent [88].

Organophosphorous insecticide/cyclodextrin complexes were coated on synthetic fibers, such as acrylic and nylon fiber to be used for moth-repellent packaging films for clothing [89].

### 2.4. Colored Plastics

A large variety of pigments or dyes are compatible with the generally used thermoplastic polymers. When the coloring component is not compatible directly with the polymer matrix (cannot be distributed homogeneously in the molten plastic), its cyclodextrin complex might be taken into account for the production of a colored packaging material.

Anthraquinone/α-CD mixed and heated with poly(vinyl chloride) pellets yields a dye masterbatch pellet for coloration of larger amount of PVC or PVC blends [90]. Rhodamine B complexed with trimethyl-β-CD and dispersed with poly(methyl-methacrylate) is used as a visible light phosphor dye plastic laser [91]. Incorporating a photochromic dye/CD complex into a transparent plastic film, it will become photochromic [92].

### 2.5. UV-filter, Anticorrosive, Antistatic Films

Various further additives can be incorporated into plastics in the form of cyclodextrin complexes:

- If the entrapped product is UV sensitive, the packaging material should contain some of the well-known, widely used UV filters, such as sodium 2-hydroxy-4-methoxybenzophenone-5-sulfonate, 2,4-dihydroxybenzophenone, or 2,2′,4,4′-tetrahydrobenzophenone [70].
- By the incorporation of antioxidants, e.g., butyl hydroxytoluene, tocopherol acetate, FeSO_4_ etc. the oxidative deterioration of the entrapped product can be decelerated [37,66,93].
- Complexation of plastic additives, such as antistatic agents and UV-absorbers with cyclodextrins and their incorporation into plastics results in products of longer durability [94].
- Antistatic polypropylene extruded films were prepared by mixing the resin pellets with cyclodextrin complexes of antistatic agents [95]. Stretch-resistant and aging-resistant plastic films were produced by adding hydroxypropyl β-CD to polyvinyl chloride–polystyrene mixture containing UV filters, antibacterial agents and plant oil [96].
- Nickel-cyclohexyl-ammonium nitrite/β-CD complex mixed with polyethylene pellets was blow-molten into a corrosion inhibiting film [97]. Rust preventing films are produced by incorporating volatile corrosion inhibitor, such as dicyclohexyl ammonium nitrite, hexamethylenetetramine or benztriazole, into thermoplastic resin, such as polypropylene, polyethylene, ethylene-vinylacetate copolymer and poly(vinyl chloride) [98].

### 2.6. Miscellaneous

Complexes of antioxidants, such as α-tocopherol and quercetin can be blended into the polymer, e.g., into low-density polyethylene before manufacturing the packaging material. These natural antioxidants have dual functions: prevent the oxidative degradation of the polymer during melt processing and protect the packaged food from oxidation during storage [66].

Edible films showing humidity triggered release were prepared by electrospinning a blend of aroma components, cyclodextrin and pullalan [99]. Edible films for packaging chewing gum contains also antioxidant, cyclodextrin and cellulose [100].

The (bio)active component might be the gaseous methylcyclopropene. When it is released slowly (from its CD complex, incorporated into the packaging–thermoplastic or paper) in a closed box which contains plant, flowers, fruits, its ethylene-antagonist effect prolongs the freshness, decelerates the ripening process of the packed products [57,98,101,102]. On the other hand, controlled postharvest ripening of fruits and vegetables can be achieved by using ethylene/α-CD complex incorporated into polymeric film or into a polymeric label used in the food container as an alternative to the injection of ethylene [31,103].

The biodegradability of the discarded packaging materials is also an important issue. The three naturally occurring cyclodextrins α-, β-, γ-CD proved to be biodegradable under laboratory controlled composting conditions [104]. The cyclodextrin derivatives have lower biodegradability than the native ones in general, individual rates depending on the type and number of substituents. For economic reasons, the easily degradable natural cyclodextrins are used in packaging.

A family of biologically degradable plastics has been developed: a biodegradable complex of a specific plastic-deteriorating agent for example, surfactant is blended into the plastic matrix [105]. Cyclodextrin protects the plastic from the action of the deteriorating agent during the application of the article. However, when the plastic article is wasted, the β-CD is broken down by microbial decomposition and the deteriorating agent is released. As the deteriorating agent acts on the plastic, the physical breakdown of the outer surface starts which goes further toward the inner parts of the plastic through cracks. This process could continue until the whole plastic article gets eroded [105].

## 3. Empty Cyclodextrins as Penetration Barriers in Packaging Films

The aim of incorporating empty cyclodextrins into a packaging film is hindering the escape of flavor/fragrance materials from the closed package or sorption of the unwanted volatile components which otherwise would penetrate into the packaged product. The aim is to decelerate both the inward and outward migration (diffusion and transmission) rate of volatile substances, from the packaging material itself, from the environment (inward) or from the packaged product (outwards). In such cases the possible highest cyclodextrin content in the film is recommended. Various CDs show different selectivity for various penetrating substances, components of solvents and other environmental volatile pollutants, e.g., cigarette smoke, exhaust gases, vapors. These volatile substances generally are compatible with the packaging material, which is permeable for them in both direction depending on the concentration difference of the permeants between the inside and outside of the package. The cyclodextrins incorporated into the packaging material, entrap easily both the penetrating volatiles, atmospheric pollutants migrating inward as well as the aroma substances escaping outward. This is the essence of the aroma-barrier packaging, some examples are listed in Table 2.

Oil-resistant packaging material was obtained by incorporating cyclodextrin in a polymer film, representing hydrophilic spots within the hydrophobic polymer matrix this way reducing the permeation of oil through the film layer [106]. By treating starch with the cyclodextrin glyucosyltransferase enzyme the so-called “raw” conversion mixture is obtained, which consists of various cyclic and a large variety of acyclic dextrins. This mixture was used as a binding layer on a cellophane film, coated with poly(vinylidene chloride). The binding layer inhibited the cracking, shrinking and peeling of the cellophane from the polyvinyl sheet. This laminated film was used to produce sausage casing [107].

The migration of model flavors, such as carvone, vanillin and diacetyl through poly(vinyl chloride) films containing 0, 1.0, 1.5 and 2.0% β-CD showed a cyclodextrin concentration-dependent penetration for the complex-forming carvone and vanillin, while diacetyl with poor complex forming ability could not penetrate through the films [123].

Incorporation of cyclodextrins into the packaging films did not change the transparency and flexibility when proper wetting agent, such as glycerol was used [114]. The barrier properties against water vapor, oxygen and carbon dioxide were slightly reduced.

**Figure 3:**
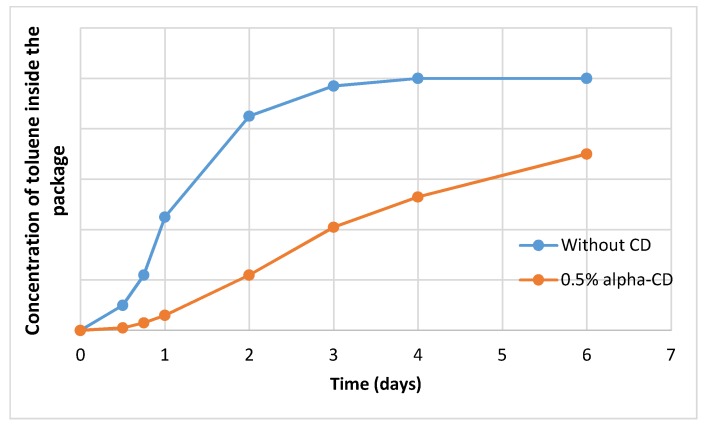
Permeation profile of a model pollutant through a common, or through a cyclodextrin-containing packaging (based on data from [110]).

The penetration of toluene vapor into a closed package was characterized by a lag-phase (the saturation of the packaging matrix) and a transmission phase and finally the saturation level (Figure 3) [110]. When CD-traps were built into the packaging film, the lag-phase was considerably longer, and the steady state phase showed a slower increase in concentration of permeant inside the package.

**Figure 4:**
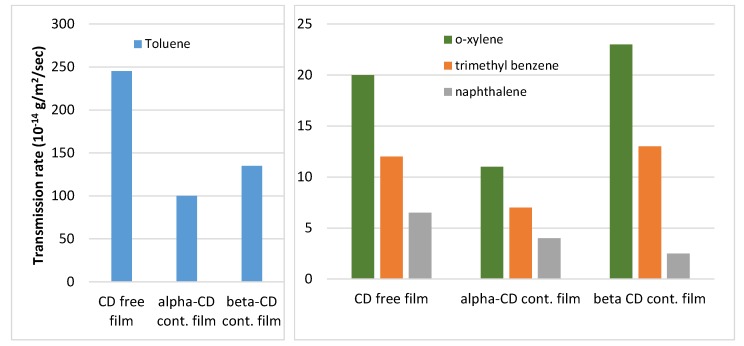
Transmission rate of several aromatic hydrocarbons through a usual, and through 0.5% α- and β-CD-containing polyethylene films (based on data from [110]).

Figure 4 illustrates the transmission rate of four penetrants through a high density (0.96 g/cm^3^) polyethylene blown film as determined by head-space gas chromatography. The amount of residual volatile components, released from a polypropylene film containing 0.5% α-CD has been reduced by 45 to 85% as compared with the CD-free film. Incorporation of 0.5% beta-cyclodextrin reduced the diffusion rate for different penetrants in average by a factor of 2.7, and the transmission rate by a factor of 3.7 [110].

The color-printed paperboard carton boxes, used for packaging, for example of cereals, release about 50 different volatile substances determined by head-space gas chromatography/mass spectrometry. The cereals were packed into closed polyethylene bags, placed into the carton box. Within a week about 2200 ng/g paperboard-released volatiles penetrated through the polyethylene bag into the cereals, but only about 1000 ng/g, if the paperboard backside (internal side) was coated with an 1:1 mixture of α- and γ-CD (0.43 g/m^2^) [110].

The same concept was used when the cholesterol sorption was studied in order to reduce this undesired component in food and cholesterol-scavenging packaging films were developed for milk [114,115]. The films also adsorbed hexanal, the oxidation product of peanut.

During heat-sealing of plastic packages a so-called breakdown smell is perceptible, which may at least partially penetrate into the packaged food product. When the packaging film contained cyclodextrin, this breakdown smell was reduced [124,125].

Incorporating cyclodextrin in polymers can reduce the migration of plasticizers as shown in the presence of β-CD in the poly(vinyl chloride) films [123]. β-CD partially modified with 3-(methacryloxy)propyl trimethoxysilane was built in the poly(vinyl chloride) structure by radical polymerization, resulting in reduced migration of dioctyl phthalate, an endocrine disrupting plasticizer [122,126]. Cyclodextrin derivatives, e.g., 2,3,6-tri-O-benzoyl β-CD are hydrophobic enough to be compatible with hydrophobic polymers, such as poly(vinyl chloride). They are mixed to the polymer before melting to result in products with decreased migration of phthalate plasticizers [127].

An important requirement for (bio)active component-releasing polymer/cyclodextrin composites will be the control of direction of the release. For example, in the case of a fungicide film used for packaging of hard cheese, to improve its shelf-life, the possible highest fraction of the volatile fungicide must be released inside the package. An insect repellent film must release its active component, contrary, outward. A rodenticide, or an UV-filter containing packaging material must not release the active component at all. The feasible solution for the oriented release is the two-layered packaging composites, consisting from a hardly penetrable support and a carrier layer, which contains the component to be released.

## 4. Conclusions

Thermoplastic-polymer-based packaging materials (films, bottles, containers, trays, etc.) are widely produced and used. Active packaging materials can be produced for specific purposes, incorporating and slowly releasing active substances (antimicrobials, insecticides, insect repellents, fragrant components, plant hormones, etc.). Application of these active substances in cyclodextrin-complexed form has several advantages:

- The loss (decomposition, volatilization etc.) of these biologically active substances at the necessary high temperature during production can be reduced. The dry cyclodextrin complexes of these substances are generally stable up to the thermal degradation temperature of the cyclodextrin (220 to 250 °C).
- The incorporation of cyclodextrin-complexed (bio)active substances into the polymer matrix do not reduce their chemical stability.
- Preparing such packaging materials, relatively small amount of the (bio)active components are distributed evenly over a large surface, to exert their effects on the surface of the packaged goods either by direct contact, or in the gas-phase within the closed package. The prerequisite of effective blending of cyclodextrin-complexed actives into the polymer phase is the compatibility of the components: less apolar polymers and less polar cyclodextrin derivatives can be successfully blended.
- The release of the complexed and polymer-incorporated (bio)active substances depends on the hydrophobicity of the matrix, permeation of water into the polymer, particle size of the complex incorporated, temperature, presence of other hydrophilic components, etc. The release must be slow to avoid the permeation of significant fraction of the (bio)active component into the packaged goods. This can be tuned by applying proper cyclodextrins.

On the other hand, when empty cyclodextrin is incorporated into the packaging material, the penetration of volatile components of the packaged good is strongly decelerated in both directions, reducing the loss of the valuable volatile flavor substances and preventing the uptake of environmental volatile contaminants.

New packaging materials must be biodegradable to avoid further environmental pollution by the biodegradation-resistant polymers (abandoned packaging materials: films, bottles, etc.). The biodegradability is a further advantage of utilization of cyclodextrins in packaging.

## Figures and Tables

**Table 1:** Cyclodextrin containing packaging materials.

Active Guest	CD	Carrier Material	Carrier Form	Application	Ref.
allyl isothiocyanate	CD	poly(ethylene-terephthalate)/poly(ethylene vinyl acetate)	film	antimicrobial food packaging	[17]
	β-CD	polyethylene	tray	antibacterial packaging for raw tuna	[18]
		natural pulp	sheet	water-absorbing antimicrobial sheet e.g., for raw tuna	[19]
		poly(vinyl alcohol)	film	antimicrobial food packaging	[20,21,22]
	triacetyl β-CD	polyethylene	film	antimicrobial	[23]
	β-CD	poly(lactic acid)	film, container	antimicrobial packaging for cheese	[12,24]
bromo-cinnamaldehyde	CD	polyethylene	film	antimicrobial or rust proof	[25]
carvacrol	β-CD	microfibrillated cellulose	paper	antibacterial packaging	[26]
		chitosan	film	antimicrobial packaging for chicken filet	[27]
	HPBCD	oxidized cellulose	film	prolonged release	[28]
catechins	CD	styrene copolymers	film	antibacterial activity and antifogging	[29]
cedar leaf oil	CD	thermoplastic resins	container	antibacterial and worm-repellent	[30]
ethylene	α-CD	poly(ethylene glycol) diacrylate	film	controlled ripening by controlled release of ethylene	[31]
essential oil	β-CD	chitosan	film	antibacterial packaging	[32]
		chitosan/poly(vinyl alcohol)	film	antifungal electrospun nanofibers	[33]
		poly(vinyl alcohol)	film	antimicrobial electrospun nanofilm	[34]
		poly(lactic acid)	film	antimicrobial electrospun nanofilm	[35]
		zein	membrane	antimicrobial electrospun composite	[36]
FeSO_4_	γ-CD	polypropylene	bottle	oxygen barrier	[37]
gallic acid	β-CD	chitosan	film	antioxidant for fatty food	[38]
geraniol	α-CD	polyethylene	cups, brushes	long-lasting fragrance	[39]
	γ-CD	poly(vinyl alcohol)	film	prolonged durability	[40]
hinokitiol	β-CD	packaging material	printing ink	freshness preservation	[41]
		polyethylene	film	fruit, vegetable antifungal packaging	[42]
		polyethylene/ethylene-vinyl acetate copolymer	film	antimicrobial food packaging	[43]
		polypropylene/polybutene	film	antimicrobial food packaging	[44]
		paper, cloth	film	antimicrobial food packaging	[45]
	CD	thermoplastic resin	film	antimicrobial food packaging	[46]
		polyolefins	packaging case	antimicrobial food pack	[47]
		packaging container	adhesive tape	antimicrobial and insect repellent	[48]
iodine	β-CD	Et-cellulose polypropylene cloth	film	sea food	[11]
isocyanate/terpene	CD	polystyrene	film	antimicrobial food packaging	[49]
		wrapping material	vacuum packaging material	antimicrobial food packaging	[50]
		polyester/vinylidene chloride/polyurethane	bag of permeable wall	antimicrobial food packaging	[51]
		starch, methyl cellulose	packaging material	edible antimicrobial packaging for candied fruits	[52]
		packaging material	coating	antimicrobial food packaging	[53]
limonene	β-CD	polyester/Al/polyethylene	container, film	orange juice, flavor improvement	[54]
		pullalan	electrospun nanofibers	active food packaging	[55]
		poly(butylene succinate)	compression molded composite films	antimicrobial food packaging	[56]
methyl-cyclopropene	α-CD	poly(vinyl alcohol)	film cardboard or plastic container, wood box	prolonged plant life and delayed ripening by inhibiting ethylene response	[57]
		polystyrene	fiber mat	humidity-triggered release	[58]
mustard oil	β-CD	cellulose sulfate	film	antimicrobial edible films and coatings	[59]
2-nonanone	β-CD	poly(lactic acid), polyethylene	film	antibacterial packaging	[60]
polyphenols, terpenes	CD	crosslinked polyethylene	coating	antibacterial, fungicide, deodorizing effect	[61]
rapeseed oil	β-CD	polypropylene	film	to separate moist/dry components of pre-packed foods (rice)	[62]
tea tree oil	β-CD	poly(ethylene oxide)	nanofiber film	antibacterial packaging	[63]
thyme oil	β-CD	alginate, caseate	film	antibacterial packaging	[64]
thymol	γ-CD	zein	nanofibrous web	antibacterial food packaging	[65]
α-tocopherol	β-CD	polyethylene	film	long-lasting antioxidant effects	[66,67]
	γ-CD	poly(lactic acid)	nanofibrous web	food packaging for meat	[68]
trans-2-hexanal	β-CD	poly(lactic acid)	sheet	antimicrobial food packaging	[14]
triclosan	β-CD, γ-CD	poly(lactic acid)	nanofibrous webs	antibacterial packaging	[69]
UV-filter	β-CD/HPBCD	poly(vinyl alcohol)	film	UV-light impermeable pack	[70]
volatile microbicides	CD	polyethylene, coated	film	antimicrobial food pack	[71]
zinc nanoparticles	triacetyl α-, β-, γ-CD	matrix-material	-	barrier material in food packaging	[72]

**Table 2:** Examples of using “empty” CD in packaging material.

CD	Carrier Material	Carrier Form	Application	Ref.
CD	polyester	beverage bottle	sorption of materials that could be extracted by the beverage	[108]
CD	non-woven cellulose web or thermoplastic polymer	fiber web, coating or laminate	permeants/contaminants traps	[109]
CD	thermoplastic paperboard	packaging material	barrier for permeation of volatiles	[110]
CD	thermoplastic polymer	sealing element, polymer liner for bottles	barrier	[111]
CD	cellulose acetate	membrane	aroma-preserving packaging of roasted, ground coffee	[112]
β-CD	polyethylene	film	elimination of development of undesired odor, discoloration	[113]
β-CD	ethylene-vinyl alcohol copolymer, poly(vinyl alcohol)	film	sorption of cholesterol	[114,115,116]
β-CD	poly(ethylenimine)		adsorption of unwanted substances	[117]
α-, β-, γ-CD	polyethylene	coating	trap for odorous components of resins and adhesives	[118]
α-, β-, γ-CD derivatives	matrix material		barrier material in food packaging, diapers	[119]
CD derivative	thermoplastic resin	film	aroma barrier	[120]
β-CD trimethyl-silylether	coatings on paperboard	film	for trapping environmental contaminants	[121]
β-CD silylated	poly(vinyl chloride)	-	reduced migration of plasticizer	[122]
